# Full Asymmetric Radiation Control Through Multi‐Channel Bound States in the Continuum

**DOI:** 10.1002/nap2.70014

**Published:** 2026-01-19

**Authors:** Boyou Wang, Yanjun Bao

**Affiliations:** ^1^ Guangdong Provincial Key Laboratory of Nanophotonic Manipulation Institute of Nanophotonics College of Physics & Optoelectronic Engineering Jinan University Guangzhou China

**Keywords:** bound states in the continuum, multi‐channel bound states in the continuum, radiation asymmetry, unidirectional guided resonances

## Abstract

Bound states in the continuum (BICs) are waves exhibiting theoretically infinite quality factors, offering a powerful mechanism for extreme light confinement in photonic structures. Although breaking vertical structural symmetry in BICs‐supporting systems can induce asymmetric radiation, the radiated power typically remains partitioned between opposing half‐spaces. Furthermore, achieving arbitrary control over the amplitude ratio and phase difference of these counter‐propagating beams presents a significant challenge, thereby limiting sophisticated beam manipulation within a single half‐space. In this work, we delve into BICs within the superwavelength regime, where photonic structures inherently support multiple diffraction orders. We systematically investigate the far‐field polarization states and associated topological properties of these individual diffraction channels. Critically, by engineering a configuration that supports two co‐propagating diffraction orders directed into the same half‐space, we demonstrate comprehensive and continuous control over the resulting unidirectional guided resonances (UGRs). Full tunability of both the directionality (spanning from −1 to 1) and the relative phase difference (spanning from −π to π) between these two co‐propagating beams is achieved. This versatile manipulation of multiple beams radiating concertedly into a specific direction opens new avenues for various advanced applications.

## Graphical abstract

Bound states in the continuum (BICs) enable extreme light confinement via high Q‐factors, but their radiation splits into half‐spaces with limited beam control. Here, in super wavelength BICs (supporting multiple diffraction orders), we design co‐propagating orders in one half‐space, achieving full tuning of UGRs’ directionality (−1 to 1) and phase (‐π to π), boosting advanced applications.

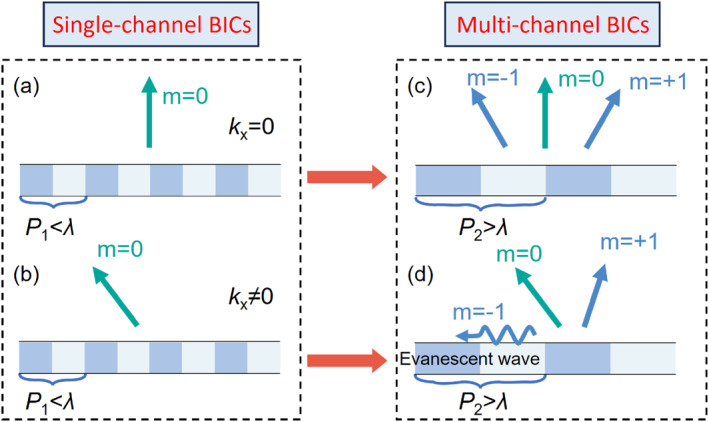

## 1 Introduction

Bound states in the continuum (BICs) are remarkable nonradiating eigenmodes embedded within the continuous spectrum of radiating modes in open photonic systems [1, 2, 3, 4, 5]. Their existence, often counterintuitive, leads to theoretically infinite quality (*Q*) factors. Such states typically manifest either through symmetry protection, which decouples the mode from accessible radiation channels [6, 7, 8, 9], or through the destructive interference of multiple leakage pathways, effectively canceling far‐field radiation [10, 11, 12, 13]. Consequently, BICs offer an unparalleled platform for achieving extreme light confinement [14, 15], enhancing light–matter interactions [16, 17, 18], and promoting the development of efficient photonic devices based on photonic crystals [19, 20, 21] and metasurfaces [22, 23, 24, 25, 26]. Over the past decade, the exploration of BICs has become a cornerstone for progress in diverse applications, ranging from low‐threshold lasing [15, 27], ultra‐sensitive sensing [28, 29, 30] to advanced nonlinear optics [31, 32, 33].

**FIGURE 1:**
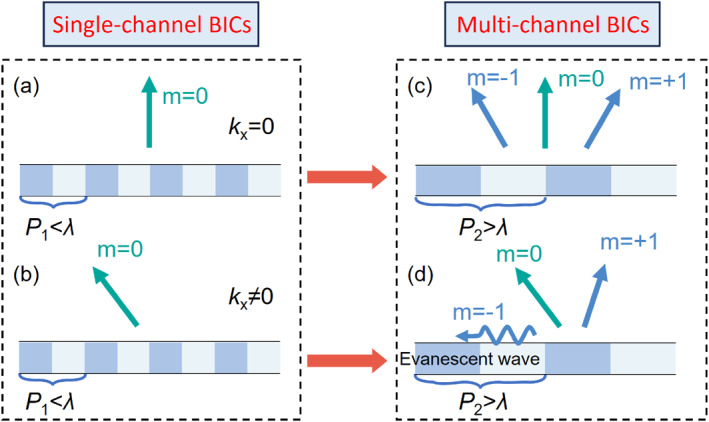
Schematic diagram of single‐ and multi‐channel BICs. (a, b) Schematic diagrams illustrating single‐channel BICs. (a) For a subwavelength period (*P*_1_ < *λ*, where *P*_1_ is the period, and *λ* is the wavelength), an eigenmode at the Γ‐point (*k*_x_ = 0) possesses a single (0th‐order) radiation channel. (b) Similarly, for an eigenmode at a non‐Γ point (*k*_x_ ≠ 0) in a subwavelength structure, only one radiation channel (0th‐order) generally propagates into the far‐field. (c) Schematic of a three‐radiation‐channel scenario occurring at the Γ‐point in a superwavelength structure (*P*_2_ > *λ*). Here, three diffraction orders (e.g., −1st, 0th, +1st) can propagate as they lie above the light line. (d) Schematic illustrating a two‐radiation‐channel scenario at a non‐Γ point (*k*_x_ ≠ 0) in a superwavelength structure (*P*_2_ > *λ*). In this configuration, one diffraction order (−1st‐order) can fall below the light line and become evanescent, while two other orders remain radiative into the same half‐space.

Historically, research concerning BICs has predominantly concentrated on photonic structures operating in the subwavelength regime [34, 35, 36], where the structural periodicity (*P*) is much smaller than the operational wavelength (*λ*) (Figure 1a). In these configurations, even for nonzero in‐plane wavevectors (*k*_x_ ≠ 0), only the zeroth diffraction order typically propagates into the far‐field, whereas higher‐order diffractions remain evanescent (Figure 1b). The far‐field polarization characteristics of such subwavelength BICs systems have been extensively mapped, revealing a rich landscape of topological features, such as polarization vortex center (*V* points) [6], circular polarized states (*C* points) [37, 38] and line of linear polarization (*L* line) [6]. Many such structures possess vertical (*σ*_z_) symmetry, resulting in radiation patterns that are identical in the upper and lower half‐spaces (e.g., superstrate and substrate). Consequently, the deliberate breaking of this *σ*_z_ symmetry has emerged as a potent strategy for realizing asymmetric radiation, whereby the power radiated upwards and downwards can be made substantially different [35, 39]. This asymmetry has been exploited for applications such as unidirectional guided resonances (UGRs) [40, 41] and asymmetric transmission devices [39, 42]. In these symmetry‐broken designs, it is common for one zeroth‐order radiation channel to approach a near‐zero radiation condition, whereas the other remains strongly radiative, or vice versa. However, a critical limitation is that the radiative power remains distributed between opposing sides of the structure. Furthermore, precisely controlling the amplitude ratio and phase difference between these two counter‐propagating beams is challenging.

In this work, we extend beyond the conventional subwavelength approach by investigating BICs in photonic structures characterized by superwavelength periodicity (*P* > *λ*). Such superwavelength structures inherently support the propagation of multiple diffraction orders on each side of the device (Figure 1c). We systematically explore the emergence of nonradiating states associated with these individual diffraction orders. By adjusting structural parameters, we induce transitions of different diffraction orders into nonradiating states. Furthermore, we investigate scenarios with nonzero in‐plane wavevectors (*k*_x_ ≠ 0). Under these conditions, specific diffraction orders (e.g., the −1st order) can become evanescent, thereby leaving only two propagating diffraction orders directed into the same half‐space, as illustrated in Figure 1d. In this two‐order co‐propagating regime, we demonstrate the ability to precisely and continuously control the UGRs. Specifically, the relative power distribution between these two co‐propagating orders can be smoothly tuned, and their relative phase difference can be adjusted across the full −π to π range. This versatile manipulation of multiple beams radiating concertedly into a specific direction is distinct from previous works and is particularly vital for applications reliant on coherent interference, such as coherent perfect absorption [39, 43] and perfect retroreflection [44], which strictly demand specific amplitude ratios and phase matching.

## 2 The Control of the Three Radiation Channels at the Γ Point

**FIGURE 2:**
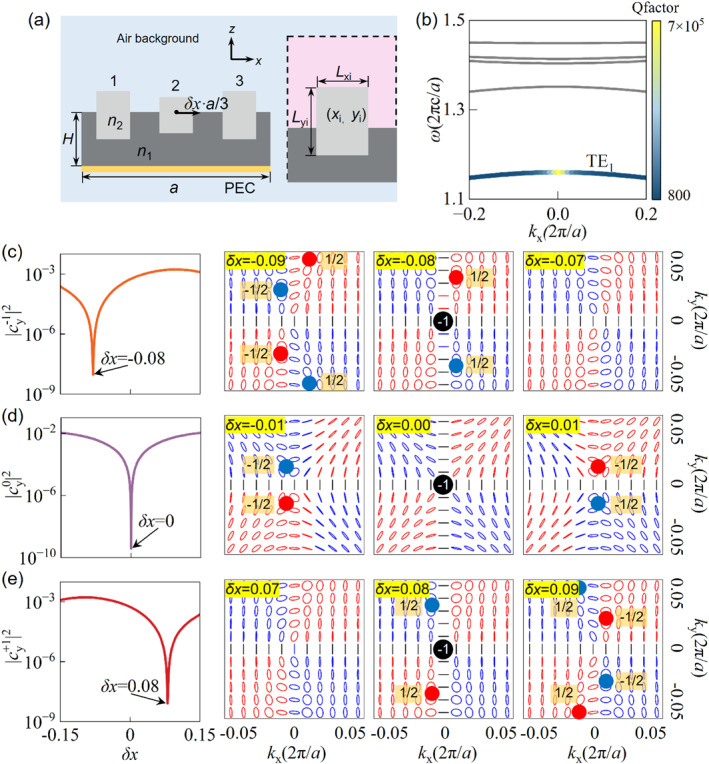
Selective suppression of three distinct radiation channels at the Γ‐point (*k*_x_ = 0) and associated topological charge dynamics. (a) Schematic of the 1D grating featuring three dielectric pillars per unit cell on a dielectric slab, backed by a PEC substrate. The inset on the right side shows the geometric details of one of the dielectric pillars. (b) Calculated TE‐polarized band structure and *Q* factor for the symmetric configuration (*δx* = 0). The color of the dispersion curve represents the quality (*Q*) factor of the eigenmodes, as indicated by the color bar. (c–e) Evolution of the Fourier normalized intensity ${\left|c_{y}^{n}\right|}^{2}$ (left panels) and the far‐field polarization states (represented by Stokes parameters or polarization ellipses in *k*‐space, right panels) of the −1st, 0th, and +1st radiation channels, respectively, as a function of the central pillar offset *δx*. Selective suppression for the −1st, 0th, and +1st radiation channels is achieved at *δx* = −0.08, *δx* = 0, and *δx* = 0.08, respectively.

Figure 2a illustrates the proposed one‐dimensional grating structure, which has a lattice constant of *a*. The structure consists of a central dielectric layer (height *H* = 0.2667*a*, refractive index *n*_1_ = 1.444), supporting an array of three dielectric pillars per unit cell, all sharing the same refractive index *n*_2_ = 3.4767. To ensure radiation is directed only upwards, a perfect electric conductor (PEC) is positioned at the bottom of the structure. The geometric parameters of the three dielectric pillars are detailed in the right inset of Figure 2a: pillar 1 has dimensions (width *L*_x1_, length *L*_y1_) = (0.1833*a*, 0.2189*a*), pillar 2 has (*L*_x2_, *L*_y2_) = (0.1852*a*, 0.1548*a*), and pillar 3 has (*L*_x3_, *L*_y3_) = (0.1833*a*, 0.2189*a*). The coordinate origin is set at the center of the substrate's upper surface. With respect to this origin, the central positions of the pillars are (*x*_1_, *y*_1_) = (−0.3333*a*, 0), (*x*_2_, *y*_2_) = (*δx*·*a*/3, −0.0323*a*) and (*x*_3_, *y*_3_) = (0.3333*a*, 0), respectively. Here, *δx* represents a tunable lateral offset for the central pillar, serving as our primary tuning mechanism. Figure 2b presents the calculated transverse electric (TE) band structure and *Q* factor along the *k*_x_‐axis for the symmetric case *δx* = 0, obtained using the finite element method (FEM). Our investigation focuses on the lowest frequency band, TE_1_. At the Γ‐point (*k*_x_ = 0, the center of the Brillouin zone), the normalized frequency (2π*c*/*a*) of this TE_1_ band exceeds 1. This condition implies that the corresponding eigenmode supports three propagating diffraction channels into the upper half‐space: the −1st, 0th, and +1st orders.

To investigate the radiation properties of the diffraction orders, we decompose the electric field of the eigenmode as follows [45]:

(1) $$E=\sum_{n}c^{n}e^{i\left(k_{x}^{\left(0\right)}+nG\right)x+ik_{y}y+ik_{z}^{\left(n\right)}z}$$

where $E=\left[E_{x},E_{y},E_{z}\right]$ represents the electric field vector, $c^{n}=\left[c_{x}^{n},c_{y}^{n},c_{z}^{n}\right]$ is the complex amplitude vector of the *n*th‐order Fourier component, *G* = 2π/*a* is the reciprocal lattice vector, $k_{x}^{\left(0\right)}\in \left[-G/2,G/2\right]$ is the in‐plane wavevector component within the first Brillouin zone, and $k_{z}^{\left(n\right)}=\sqrt{k_{0}^{2}-k_{y}^{2}-{\left(k_{x}^{\left(0\right)}+nG\right)}^{2}}$ is the out‐of‐plane component of *n*th‐order wave vector. The nature of $k_{z}^{\left(n\right)}$ determines whether the component is radiative (real $k_{z}^{\left(n\right)}$) or nonradiative (imaginary $k_{z}^{\left(n\right)}$). For each radiative *n*th‐order Fourier component, its polarization state in the far‐field (momentum space, or *k*‐space) can exhibit topological singularities. The topological charge $q^{\left(n\right)}$ of such a singularity is defined as follows:

(2) $$q^{\left(n\right)}=\frac{1}{2\pi }\oint_{L}dk_{∥}\cdot \nabla_{k_{∥}}\psi^{\left(n\right)}\left(k_{∥}\right)$$

where *L* is a counterclockwise closed loop around a polarization singularity in momentum space, $k_{∥}=\left(k_{x},k_{y}\right)$ is the in‐plane wave vector. $\psi^{\left(n\right)}\left(k_{∥}\right)=\frac{1}{2}\arg\left(S_{1}^{\left(n\right)}+iS_{2}^{\left(n\right)}\right)$ is the orientation angle of the major axis of the polarization ellipse. This ellipse is determined by the transverse components determined by $\left[c_{x}^{n},c_{y}^{n}\right]$ of the *n*th‐order Fourier coefficient, and $S_{i}^{\left(n\right)}$ are the corresponding Stokes parameters.

To ensure a consistent comparison of radiation strengths, the eigenmodes are normalized such that the total electromagnetic energy stored within the unit cell is unity. Under this normalization, the squared magnitude of the Fourier coefficients is directly proportional to the radiation leakage rate into that specific channel. Consequently, a low value of ${\left|c_{i}^{n}\right|}^{2}$ implies weak coupling to the continuum, corresponding to a high‐*Q*, quasi‐BIC state. The left panels of Figure 2c–e illustrate the calculated normalized intensity ${\left|c_{y}^{n}\right|}^{2}$ of the three diffraction Fourier components as a function of the tuning parameter *δx*. These results clearly demonstrate that the −1st, 0th, and +1st diffraction orders can be individually and completely suppressed at specific *δx* values of −0.08, 0 and 0.08, respectively. At these specific *δx* values, radiation is forbidden with respect to that particular diffraction channel, and consequently, radiation is funneled exclusively through the remaining two radiative orders. The vanishing of a specific diffraction order is intimately linked to topological transformations occurring in its *k*‐space polarization distribution (right panels of Figure 2c–2e). For instance, consider the −1st radiation channel (Figure 2c): as *δx* approaches −0.08, two *C* points, each typically carrying a topological charge of −1/2, are observed to merge at the *k*‐space origin (***k***_∥_ = (0, 0)). This merging event leads to the formation of a *V* point, which is characterized by an integer topological charge of −1, and signifies the complete suppression of radiation into this −1st channel. Moving *δx* away from this critical value causes the *V* point to split back into *C* points, thereby restoring the radiative nature of the channel. A similar mechanism underpins the suppression of the 0th radiation channel (Figure 2d): as *δx* approaches 0, the merging of *C* points results in the formation of a *V* point at ***k***_∥_ = (0, 0), extinguishing the 0th order radiation. Owing to the initial *C*_2_ rotational symmetry of the structure when *δx* = 0 (for pillars 1 and 3 being identical and symmetrically placed), the topological charge evolution for the +1st radiation channel (Figure 2e) exhibits analogous behavior, with its suppression occurring at *δx* = +0.08, also marked by *V* point formation. It is crucial to note that all such generation, annihilation, and transformation events involving these topological charges strictly adhere to the principle of topological charge conservation [46] within the *k*‐space representation of each individual radiation channel. Consequently, *C* points of the same charge can merge to form higher‐order singularities such as *V* points. It is noteworthy that although the structure possesses *C*_2_ symmetry at *δx* = 0, which successfully suppresses the 0th‐order radiation, the ± 1st‐order channels remain radiative. This phenomenon is attributed to the fact that the eigenmode at the Γ‐point is odd‐symmetric. The 0th‐order plane wave functions as an even‐symmetric mode, creating a symmetry mismatch that suppresses radiation in this channel. Conversely, when the wavefields of the ± 1st‐order diffraction channels are superimposed with a π phase difference, they form an odd‐symmetric far‐field distribution. This pattern perfectly matches the symmetry of the eigenmode, thereby enabling the ± 1st‐order channels to sustain radiation even under the condition of *C*_2_ symmetry.

## 3 The Control of the Two Co‐Propagation Radiation Channels at a Non‐Γ Point

**FIGURE 3:**
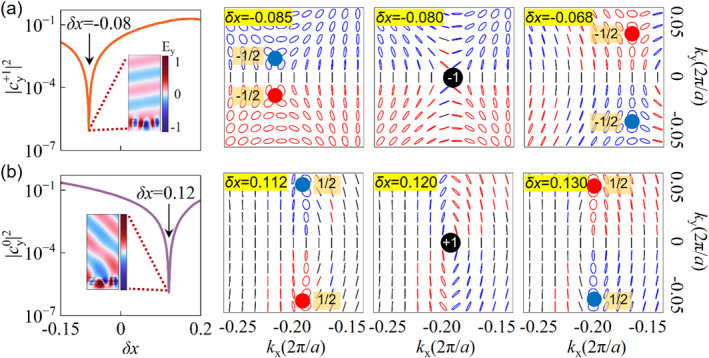
Selective suppression of two co‐propagating radiation channels at a non‐Γ point (*k*_x_ = −0.194 (2π/*a*)). (a, b) Simulated Fourier normalized intensity ${\left|c_{y}^{n}\right|}^{2}$ (left panels) and corresponding far‐field polarization distributions in *k*‐space (right panels) for the two radiative orders. (a) For the +1st channel (*n* = +1), suppression occurs at *δx* = −0.08. The inset displays the simulated electric field distribution, indicative of radiation predominantly into the 0th‐order channel. (b) For the 0th channel (*n* = 0), suppression occurs at *δx* = 0.12. The inset shows the electric field pattern corresponding to radiation primarily into the +1st‐order channel.

The analysis thus far has concentrated on the Γ‐point (*k*_x_ = 0), where three diffraction orders are radiative. We now shift our attention to an off‐normal scenario, specifically by setting the in‐plane wavevector to *k*_x_ = −0.194 (2π/*a*). At this nonzero *k*_x_, the −1st diffraction order becomes evanescent as it falls below the light line. Consequently, only the 0th and +1st diffraction orders remain radiative. Significantly, both of these orders now propagate into the same (upper) half‐space, creating a co‐propagating two‐channel system. For this two‐channel configuration, the structural parameters were refined as follows: pillar dimensions (*L*_x1_, *L*_y1_) = (0.1833*a*, 0.2236*a*), (*L*_x2_, *L*_y2_) = (0.1833*a*, 0.1448*a*), and (*L*_x3_, *L*_y3_) = (0.1833*a*, 0.215*a*), and their central positions (*x*_1_, *y*_1_) = (−0.3333*a*, 0.0043*a*), (*x*_2_, *y*_2_) = (*δx*·*a*/3, −0.0313*a*), and (*x*_3_, *y*_3_) = (0.3333*a*, 0), respectively. By varying the offset parameter *δx* from −0.15 to 0.2, we observe the selective suppression of these two co‐propagating radiation orders, as detailed in Figure 3. When one of these radiation orders is suppressed to form a BIC‐like state for that channel, the other remains strongly radiative. This effectively achieves high UGRs, where the energy of the eigenmode is channeled predominantly into a single diffraction order, both propagating within the same half‐space. The inset panels, displaying simulated electric field distributions, provide clear visual confirmation of these distinct UGRs phenomena.

To elucidate the underlying mechanism responsible for the vanishing of these individual co‐propagating diffraction orders, we again turn to an analysis of the topological properties of their far‐field polarization patterns in *k*‐space. As illustrated in Figure 3a for the +1st radiation channel, tuning *δx* to approximately −0.08 causes two *C* points, each typically possessing a topological charge of −1/2, to converge and merge at the center of its *k*‐space map (***k***_∥_ corresponding to the +1st order direction). This interaction culminates in the formation of a *V* point, which carries a net integer topological charge of −1, thereby leading to the suppression of the +1st radiation channel. Conversely, for the 0th radiation channel (Figure 3b), as *δx* approaches approximately 0.12, two *C* points, each with a topological charge of +1/2, undergo a similar merging process at the center of its *k*‐space map. This generates a *V* point with a net charge of +1, consequently extinguishing the 0th radiation channel. It is important to emphasize that although these topological features (*C* points and *V* points) are associated with the same underlying eigenstate (at a fixed overall wavevector ***k*** and frequency *ω*), they manifest independently within the *k*‐space polarization representation of each different radiation channel. Therefore, topological singularities belonging to, for example, the 0th diffraction order are generally independent of those belonging to the +1st diffraction order and do not directly interact (merge or annihilate) with each other.

## 4 Full Tunability of Both the Directionality and the Relative Phase Difference

**FIGURE 4:**
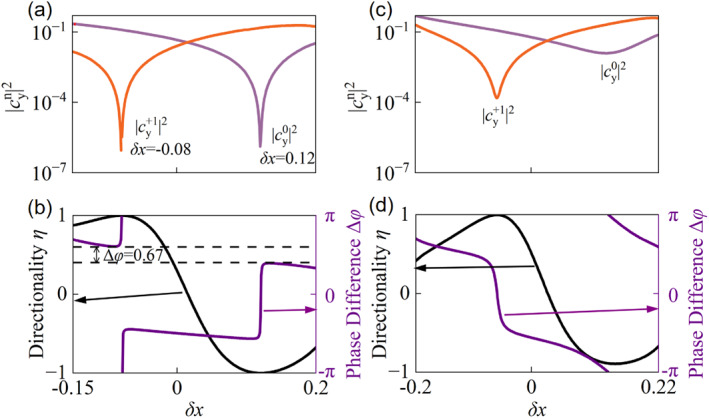
Comprehensive control over directionality and relative phase for two co‐propagating radiation channels at *k*_x_^(0)^ = −0.194 (2π/*a*). (a) Fourier radiated intensities of the 0th and +1st diffraction channels versus *δx*, using parameters from the Figure 3, illustrating conditions that yield strong UGRs. Minima for the +1st and 0th channel intensities occur near *δx* = −0.08 and *δx* = 0.12, respectively. (b) Calculated directionality *η* and relative phase difference Δφ versus *δx* for this initial parameter set. The phase difference Δφ exhibits a discontinuity (gap of ≈ 0.67 radians), precluding full 2π coverage. (c) Fourier radiated intensities of the 0th and +1st channels versus *δx* following geometric parameter refinement. (d) Directionality *η* and relative phase difference Δφ versus *δx* for the refined parameters. Although the range of *η* is slightly diminished, the relative phase Δφ now varies continuously over the complete −π to π range, demonstrating full phase agility.

Beyond achieving conditions of nearly perfect UGRs (where one channel is almost completely suppressed), the meticulous tuning of structural parameters grants a more sophisticated level of control, allowing for the fine adjustment of both the relative amplitudes and phases of the two co‐propagating beams. To quantify this nuanced control, we introduce the directionality parameter *η,* defined as $\eta =\left({\left|c_{y}^{0}\right|}^{2}-{\left|c_{y}^{+1}\right|}^{2}\right)/\left({\left|c_{y}^{0}\right|}^{2}+{\left|c_{y}^{+1}\right|}^{2}\right)$ and the relative phase difference $\Delta \varphi =\arg(c_{y}^{0})-\arg(C_{y}^{+1})$ between the 0th ($c_{y}^{0}$) and +1st ($c_{y}^{+1}$) diffraction orders. Figure 4a plots the Fourier intensities of these two channels as a function of *δx* using the parameters from that shown in Figure 3. The pronounced intensity minima observed for the +1st and 0th channels (near *δx* = −0.08 and *δx* = 0.12, respectively) decrease to levels approaching 10^−7^, confirming the strong UGRs predominantly into either the 0th or the +1st channel, as discussed previously. The corresponding calculated directionality *η* and relative phase difference Δφ are plotted in Figure 4b as a function of *δx*. This figure clearly demonstrates that the directionality *η* can be continuously tuned across its entire theoretical range, from approximately −1 (indicating power predominantly channeled into the +1st order) to +1 (power predominantly in the 0th order). However, for this set of initial parameters, achieving complete 2π coverage of the relative phase difference proves elusive, evidenced by a noticeable phase gap of approximately 0.67 radians. Additionally, abrupt phase jumps are observed as the directionality approaches ± 1. These discontinuities arise because the intensity of one diffraction channel vanishes (radiation nulls) at these limits, rendering its phase undefined (a phase singularity).

To attain comprehensive 2π phase control, we undertook a further refinement of the geometric parameters, specifically adjusting *L*_y2_ to 0.1419*a* and *y*_2_ to −0.0355*a*, whereas other parameters remained as that in Figure 3. The resultant intensity profiles of the two diffraction orders as a function of *δx*, post‐refinement, are displayed in Figure 4c. Notably, the intensity minima in this scenario are elevated from near‐zero values. This indicates a strategic departure from perfect unidirectionality for either channel, a compromise made to facilitate smoother and more complete phase transitions. The corresponding directionality *η* and relative phase difference Δφ for these refined parameters are presented in Figure 4d. With this optimized geometry, the achievable range of the directionality parameter *η* is marginally reduced, although it still effectively spans almost the entire −1 to +1 range. Most importantly, the relative phase difference Δφ between the two co‐propagating radiation channels can now be continuously and smoothly controlled across the entire −π to π range simply by varying the offset *δx*. This demonstrated capability for continuous full‐range manipulation of both the directionality, and the relative phase difference of two beams co‐propagating into a single half‐space offers highly versatile and practically feasible pathways for a broad spectrum of advanced optical applications.

## 5 Conclusion

In summary, we have systematically investigated the far‐field polarization characteristics of bound states in the continuum manifested in superwavelength photonic structures, which inherently support multiple radiation channels. Through precise engineering of geometric parameters, we have demonstrated the sequential suppression of individual radiation channels. This suppression is fundamentally linked to the controlled manipulation—generation, merging, and transformation—of topological polarization singularities, specifically *C* points and *V* points, within the momentum‐space representation associated with each distinct channel. Building upon this understanding, we have proposed and numerically validated a robust strategy for achieving comprehensive control over two optical beams that co‐propagate into a single designated half‐space. This control encompasses the ability to tune the directionality (relative power distribution) continuously from approximately −1 to +1. More significantly, we have realized full continuous −π to π mastery over the relative phase difference between these two co‐propagating beams. Although this work focuses on the theoretical mechanism, the robust topological nature of these phenomena suggests feasibility for experimental realization. Our findings provide a precise design blueprint for controlling multi‐channel coherence, paving the way for practical implementations in coherent beam shaping and signal processing in future works.

## Author Contributions

**Boyou Wang:** validation, data curation, writing – original draft. **Yanjun Bao:** conceptualization, writing review and editing, supervision.

## Data Availability

Data sharing not applicable to this article as no datasets were generated or analyzed during the current study.
